# Performance evaluation between two automated biochemical analyzer systems: Roche Cobas 8000 and Mindray BS2000M

**DOI:** 10.5937/jomb0-34328

**Published:** 2022-07-29

**Authors:** Mingxing Chen, Simeng Qin, Sitao Yang, Huaping Chen, Liuyi Lu, Xue Qin

**Affiliations:** 1 First Affiliated Hospital of Guangxi Medical University, Department of Clinical Laboratory, China; 2 Ministry of Education, Guangxi Medical University, Key Laboratory of Early Prevention and Treatment for Regional High-Incidence-Tumor, Nanning, Guangxi, P.R. China

**Keywords:** Analytical techniques and equipment, validation, Roche Cobas 8000, Mindray BS2000M, comparison, analitičke tehnike i oprema, validacija, Roche Cobas 8000, Mindray BS2000M, poređenje

## Abstract

**Background:**

The values of biomarkers play a central role in routine clinical decision-making. Whereas the performance of different automated chemical analyzers remains unclear. To determine the performance of different platforms, we compared the consistency and accuracy between Roche Cobas 8000 and Mindray BS2000M.

**Methods:**

A total of 1869 remaining serum samples were collected. CK, LDH-1, RBP, Cys-C, IgA, IgM, and IgG were assessed using paired t-test, Passing-Bablok regression analysis, and Bland-Altman analysis according to CLSI EP5-A3.

**Results:**

There were significant differences in the average bias of all items between the two machines (P<0.001). Because the 95% confidence interval of intercept A included 0, CK, LDH-1, Cys-C and IgG did not show systematic error in Passing-Bablok regression analysis. The confidence interval of 95% of the slope B in IgM contained 1, and there was no difference in the two measurements in IgM. Except for IgA, the r values and correlation coefficient of all items were higher than 0.91, which showed that the correlation and consistency were good. The Bland-Altman analysis showed that two instruments had more than 95% of the points apart from CK, LDH-1, and IgA.

**Conclusions:**

It can be considered that the two instruments have good correlation and consistency in CK, LDH-1, RBP, Cys-C, IgM, and IgG, and the two instruments are interchangeable and can replace each other.

## Abstract (sr)

**Uvod:**

Vrednosti biomarkera igraju centralnu ulogu u rutinskom kliničkom donošenju odluka. S druge strane, performanse različitih automatizovanih hemijskih analizatora ostaju nejasne. Kako bismo utvrdili performanse različitih platformi, izvršeno je upoređivanje konzistentnosti i tačnosti između analizatora Roche Cobas 8000 i Mindray BS2000M.

**Metode:**

Prikupljeno je ukupno 1869 preostalih uzoraka seruma. Vrednosti CK, LDH-1, RBP, Cys-C, IgA, IgM i IgG su odre|ene korišcenjem uparenog t-testa, Passing-Bablok regresije i Bland-Altmanove analize prema CLSI EP5-A3.

**Rezultati:**

Pokazale su se značajne razlike u prosečnoj pristrasnosti svih stavki između dve mašine (P < 0,001). Pošto je interval poverenja od 95% preseka A uključivao 0, CK, LDH-1, Cys-C i IgG nisu pokazali sistematsku grešku u Passing-Bablok regresionoj analizi. Kod intervala poverenja od 95% nagiba B u IgM koji je sadržao 1, nije bilo razlike u dva merenja u IgM. Osim za IgA, r vrednosti i koeficijent korelacije kod svih stavki su bili veći od 0,91, što je pokazalo dobru korelaciju i konzistentnost. Bland-Altmanova analiza je pokazala da su dva instrumenta imala više od 95 procentna poena osim za CK, LDH-1 i IgA.

**Zaključak:**

Može se reći da dva instrumenta imaju dobru korelaciju i konzistentnost u pogledu CK, LDH-1, RBP, Cys-C, IgM i IgG, te se mogu koristiti bez razlike.

## Introduction

The biomarkers in clinical laboratories have played a key role in medical decisions for patients' diagnosis, treatment, and prognosis [1] [2] [3] [4]. Therefore, the results tested by diagnostic machines must be more precise, accurate, sensitive, and specific. Technological advances have greatly improved the development of laboratory medicine and met the growing demands in routine biochemistry analysis, such as high throughput analysis of multi-parameters, leading to the increasing use of new analyzers. Implementing an automation analyzer in laboratory diagnostics provides advantages and convenience for requiring a high degree of value with precision and accuracy [5]. However, the detected results of the same samples by different machines are sometimes inconsistent. For example, some common clinical chemistry analytes have shown that comparable problems still exhibited unacceptable or suboptimal bias compared to the true value [6]. Imprecise or incorrect results might lead to immeasurably serious consequences for patients, clinicians, and even the entire health care system. Hence, emphasis should be placed on examining the standardized protocol [7].

However, it remains controversial whether the values of the different equipment in an identical medical laboratory from the same specimen may be inconsistent and not the same according to the standardized operating procedures. It is high time that the emphasis is placed on creating different reference intervals for different machines. Recently, most previous studies have compared automated hematology analyzers about Beckman Coulter, Sysmex, and Mindray [8] [9] [10] [11], automated hemostasis analyzer between Sysmex and Atellica [12], automated bacterial identification, and drug sensitivity analyzer between GENECUBE and Vitek [13]. However, some research has studied other different automated chemistry analyzers, such as Abbott, Roche, Beckman Coulter, and Hitachi [6] [14] [15].

Nikolac Gabej et al. [14] recently focused on three parameters about hemoglobin and bilirubin intralipid in Abbott, Roche, and Beckman Coulter. Having included 21 items, Lim et al. [6] emphasized liver and kidney function and blood lipid. Though Leitner-Ferenc et al. [1] study underlined the reference intervals on the Roche Cobas 8000 platform based on the Clinical and Laboratory Standards Institute (CLSI), their study only focused on gender difference. As we all know, automated chemistry analyzers can detect organ functions and all kinds of metabolites. These published studies have indicated excellent performance in the precision and accuracy of automated biochemical analyzers. Mindray BS2000M, a new generation automated chemistry analyzer, is a high test, less reaction volume, and multi-wavelengths system. None of the studies have focused on the performance and evaluation of Mindray BS2000M. Moreover, none of these studies have defined the performance at low, normal, and high concentration. Therefore, we designed the present study and paid attention to the differences in myocardial enzyme, kidney function, and immuno globulin in Roche and Mindray. As far as we know, we are first to compare the two machines' performance. For myocardial enzymes, such as creatine kinase (CK), which appears very early after the attack of acute myocardial infarction (AMI), its sensitivity reaches 98% in the diagnosis after the onset of the disease of AMI. Moreover, a previous study revealed that patients with high CK had a worse prognosis [16]. Another enzyme, lactate dehydrogenase-1 (LDH-1), owing to being increased in blood 5~10 hours after AMI, was also treated as an early biomarker of AMI [16]. As mentioned, many studies have shown the discrepancies of common kidney biomarkers, for instance, creatinine, blood urea nitrogen, and uric acid, between different measurements. We focused on other markers that were more sensitive and specific. Retinol binding protein (RBP), which can remain stable in acid urine and quickly appear after an early renal proximal injury, is considered to be a reliable and sensitive parameter for kidney injury [17]. Cystatin-C (Cys-C) improves the risk classification of patients with chronic kidney disease, death, cardiovascular disease [3], and end-stage renal disease [18]. As the effector molecules of the adaptive humoral immune system, high or low levels of immunoglobulins cause an allergic reaction or immunodeficiency diseases. Since immunoglobulin A (IgA) can limit antigen access to host tissues, it was referred to as the mucosal barrier in immune exclusion and shed light on the importance of regulating food allergen sensitization [19]. At the same time, the patients with Crohn's disease or ulcerative colitis also showed that serum IgA in blood was elevated [20]. While for common variable immunodeficiency and primary immunodeficiency diseases, the level of IgA of patients may be deficient [21]. Immunoglobulin M (IgM), involved in both immune protection and immunoregulatory functions, is treated as the first line of humoral defense against pathogens [22]. Reducing IgM might increase the risk of infection, exacerbate autoimmunity as well as atherosclerosis [23]. High immunoglobulin G (IgG) helps to diagnose autoimmune hepatitis [24] and IgG in cerebrospinal fluid (CSF), which is useful for the diagnosis of multiple sclerosis [15]. In addition to the mean bias in the two instruments, test characteristics related to consistency and correlation in two measurements were investigated.

Measurement of laboratory analytical errors falls into two main categories, systematic error and random error. Systematic errors are predictable problems that influence observations consistently in one direction, while random errors are more unpredictable. Sources that contribute to uncertainty may include samples, calibrators, reference materials, input quantities, equipment, and environmental conditions.

## Materials and Methods

### Samples

A total of 1869 remaining serum samples were collected from outpatients and inpatients at the Second Affiliated Hospital of Guangxi Medical University from July 2019 to October 2019 for diagnostic accuracy. All samples were tested within 2 hours after centrifugation of 4000 g for 5 minutes. Specimens that could not be tested immediately were refrigerated at 4 after centrifugation, and tests were completed within 24 hours. Samples must be thawed to room temperature and mixed thoroughly after refrigeration. After being tested on Cobas 8000 c702 (Roche, Basel, Switzerland), those serum samples were immediately tested on Mindray BS2000M (Mindray Bio-Medical Electronics Co., Ltd, Shenzhen, China) to guarantee the consistency of time and the accuracy of results. Those samples were categorized as being of abnormally high, abnormally low, or normal value. This study was approved by the Ethics Committee of the Second Affiliated Hospital of Guangxi Medical University.

### Reagents

All of the procedures were carried out according to the manufacturer's protocols. In brief, CK tested by Cobas 8000, and Mindray BS2000M used colorimetry and phosphocreatine substrate method, respectively. LDH-1tested by Cobas and Mindray used the rate method and lactic acid substrate method, respectively. Latex immunoturbidimetry by using Cobasreagents was used for RBP, Cys-C analysis, while they were examined by latex enhanced immunoturbidimetry in Mindray. For IgA, IgM, and IgG, all were detected by immunoturbidimetry in two automatic analyzers. All methods in seven parameters are summarized in Table 1.

**Table 1:** Characteristics of the compared methods between Cobas 8000 and Mindray BS2000M.

Parameter	Cobas 8000 method	Mindray BS2000M<br>method
CK	Colorimetry	Creatine phosphate<br>substrate method
LDH-1	Rate method	Lactic acid substrate<br>method
RBP	Latex immunotur-<br>bidimetry	Latex enhanced<br>immunoturbidimetry
Cys-C	Latex immunotur-<br>bidimetry	Latex enhanced<br>immunoturbidimetry
IgA	Immunoturbidimetry	Immunoturbidimetry
IgM	Immunoturbidimetry	Immunoturbidimetry
IgG	Immunoturbidimetry	Immunoturbidimetry

### Quality control

All reagents, quality control products, and calibration products were original reagents that matched with the machine. The instrument was calibrated according to the manufacturer's guidelines using calibration samples provided by the manufacturer. High, normal, and low control samples were run every day to monitor the system's performance according to the National Laboratory Accreditation Board (NABL) guideline and CLSI EP5-A3 [25]. To evaluate the quality of our results from two machines, two levels of control in seven parameters were detected every time, including Lot 32419602 and 32419602 in CK and LDH-1, Lot 1293uN, and 983uE in RBP and Cys-C, and Lot 48902 and 48903 in IgA, IgM, and IgG. The coefficient of variation of quality control in all parameters was less than 10% which means that the results of quality control were in control. There was nothing unusual in control, which demonstrates that the quality of controls was acceptable. Then the serum samples were tested according to the manufacturer's instruction and strictly followed standard operating procedure.

### Statistical analysis

All statistical analyses were performed using SPSS 20.0 (SPSS Inc., Chicago, IL, USA) and MedCalc v18.2.1 (Ostend, Belgium). The paired t-test was used to compare the mean bias of results in two instruments. Bland-Altman plot [26] [27] was used to evaluate the consistency of the two machines. Passing-Bablok regression analysis [28] was used to evaluate the regression equation and the correlation of the two instruments. If the 95% confidence interval (CI) of intercept A does not contain 0, there are systematic errors in the two instruments. The slope B was used to measure the difference in the ratio between the two instruments. The 95% CI for slope B did not include 1, which means that there are a few differences between the two methods. The Cusum test for linearity was used to test the applicability of the Passing-Bablok regression. If *P*<0.05, it indicates that there is no linear relationship between the two apparatuses, so this method is not applicable. When the correlation coefficient *r* is lower than 0.4, the correlation degree is low. If *r* is more than 0.4 but lower than 0.7, the correlation degree is moderate. If *r* is higher than 0.7, the correlation degree is high. All comparison with *P*-value <0.05 was considered statistically significant.

## Results

### Descriptive analysis of different methods

As shown in Table 2, the IgG in Cobas 8000 had a minimum CV value of 2.64%, while CK in Cobas 8000 reached 7.10%. However, all CVs of the parameter in the two instruments were lower than 10%. The paired t-test was performed, and the results revealed a statistically significant difference in all items (both *P*<0.001). All methods of different items between the two platforms were summarized in Table 1.

**Table 2:** Evaluation of the imprecision between the two measurements. Min: minimum, Max: maximum, CV: coefficient of variation

Parameter (units)	Cases	Instrument	Min	Max	Mean	CV (%)	P
CK (U/L)	241	Cobas 8000	11	1026	147.5	7.10	<0.001
		Mindray BS2000M	25	1000	159.44	6.99	
LDH -1 (U/L)	261	Cobas 8000	25	572	97.48	4.79	<0.001
		Mindray BS2000M	10	500	85.3	4.83	
RBP (mg/L)	275	Cobas 8000	7.9	157.5	44.19	3.47	<0.001
		Mindray BS2000M	10	150	38.63	4.14	
Cys-C (mg/L)	272	Cobas 8000	0	10	1.49	5.17	<0.001
		Mindray BS2000M	0.4	6.34	0.971	5.84	
IgA (g/L)	311	Cobas 8000	0.06	8.61	2.8654	3.72	<0.001
		Mindray BS2000M	0.4	6.06	2.6767	3.40	
IgM (g/L)	271	Cobas 8000	0.056	4.02	1.2744	4.71	0.001
		Mindray BS2000M	0.35	3.6	1.3206	4.43	
IgG (g/L)	238	Cobas 8000	1.99	36	12.8547	2.64	<0.001
		Mindray BS2000M	1.03	27.6	10.501	2.81	

### Comparison methods

Based on the clinical significance of these parameters level, serum samples were divided into two levels (low and high level) and three levels (low, normal, and high level). Three of seven items (LDH-1, RBP, and Cys-C) and four of seven items (CK, IgA, IgM, and IgG) were divided into either two or three levels according to the clinical reference range. All subgroups of these parameters are shown in Table 3.

**Table 3:** Spearman rank correlation, Bland-Altman plot analysis and correlation coefficient in the two systems. LOA: limit of agreement, CC: correlation coefficient, CI: confidence intervals

Parameter	Group	N	r	Average bias (95%CI)	LOA	CC (95%CI)	P
CK	All	241	0.995	-11.938 (-13.968 to -9.907)	(-43.303 to 19.428)	0.997 (0.997 to 0.998)	<0.001
	7–40 (U/L)	65	0.807	-3.846 (-4.636 to -3.056)	(-10.095 to 2.402)	0.955 (0.927 to 0.972)	<0.001
	40–300 (U/L)	127	0.994	-7.937 (-9.175 to -6.699)	(-21.756 to 5.882)	0.997 (0.995 to 0.998)	<0.001
	>300 (U/L)	49	0.975	-33.041 (-39.782 to -26.300)	(-79.040 to 12.958)	0.933 (0.884 to 0.962)	<0.001
LDH-1	All	261	0.956	12.180 (10.053 to 14.307)	(-22.020 to 46.380)	0.977 (0.971 to 0.982)	<0.001
	<90 (U/L)	125	0.910	7.288 (6.190 to 8.386)	(-4.873 to 19.449)	0.933 (0.905 to 0.952)	<0.001
	≥90 (U/L)	136	0.934	16.677 (12.852 to 20.501)	(-27.522 to 60.874)	0.898 (0.860 to0.927)	<0.001
RBP	All	275	0.910	5.579 (4.635 to 6.524)	(-10.019 to 21.178)	0.980 (0.974 to 0.984)	<0.001
	<70 (U/L)	218	0.913	5.845 (5.299 to 6.391)	(-2.167 to 13.856)	0.962 (0.950 to 0.970)	<0.001
	≥70 (U/L)	57	0.459	4.563 (0.4200 to 8.706)	(-26.042 to 35.169)	0.886 (0.814 to 0.932)	<0.001
Cys-C	All	272	0.981	0.520 (0.475 to 0.564)	(-0.208 to 1.246)	0.954 (0.942 to 0.964)	<0.001
	<1.03 (mg/L)	119	0.618	0.313 (0.300 to 0.327)	(0.168 to 0.459)	0.770 (0.686 to 0.835)	<0.001
	≥1.03 (mg/L)	153	0.981	0.679 (0.611 to 0.748)	(-0.157 to 1.516)	0.963 (0.950 to 0.973)	<0.001
IgA	All	311	0.851	0.189 (0.107 to 0.271)	(-1.252 to 1.630)	0.935 (0.919 to 0.948)	<0.001
	<0.82 (g/L)	57	0.145	-0.220 (-0.344 to -0.095)	(-1.139 to 0.700)	0.839 (0.740 to 0.902)	<0.001
	0.82–4.53	188	0.957	0.070 (0.041 to 0.099)	(-0.328 to 0.468)	0.983 (0.977 to 0.987)	<0.001
	>4.53 (g/L)	67	0.089	0.867 (0.560 to 1.174)	(-1.601 to 3.334)	0.605 (0.426 to 0.738)	<0.001
IgM	All	271	0.950	0.046 (-0.073 to -0.020)	(-0.481 to 0.389)	0.981 (0.976 to 0.985)	<0.001
	<0.46 (g/L)	68	0.436	-0.058 (-0.082 to -0.034)	(-0.255 to 0.139)	0.829 (0.736 to 0.891)	<0.001
	0.46–3.04	174	0.912	-0.071 (-0.097 to -0.044)	(-0.421 to 0.279)	0.962 (0.950 to 0.972)	<0.001
	>3.04 (g/L)	29	0.251	0.129 (-0.049 to 0.307)	(-0.787 to 1.045)	0.695 (0.441 to 0.846)	<0.001
IgG	All	238	0.904	2.354 (2.139 to 2.569)	(-0.945 to 5.652)	0.947 (0.932 to 0.958)	<0.001
	<7.51 (g/L)	46	0.737	0.793 (0.569 to 1.018)	(-0.688 to 2.274)	0.861 (0.761 to 0.921)	<0.001
	7.51–15.60	127	0.751	2.343 (2.139 to 2.546)	(0.0712 to 4.615)	0.860 (0.807 to 0.900)	<0.001
	>15.60 (g/L)	65	0.739	3.479 (2.960 to 3.999)	(-0.633 to 7.592)	0.820 (0.720 to 0.886)	<0.001

The comparison between seven items of two instruments was carried out using Passing and Bablok regression analysis and Bland-Altman plots. The results of this statistical analysis are shown in Table 3 and Table 4. A high correlation was obtained for analysis compared with two instruments for most parameters in all results but not subgroups in six items (*r* ranging from 0.904 to 0.995) except for IgA (*r*=0.857) by Spearman rank correlation analysis. However, the high level of IgA (>4.53 g/L) between the two instruments showed little correlation (*r*=0.089). Moreover, there was a high correlation between 7 parameters in the two machines according to correlation coefficient (CC) results. All CC of items were more than 0.7, whether the items had low, moderate, and high values, except when IgA was more than 4.53 g/L (CC: 0.605, 95%CI 0.426-0.738) (Table 3). All correlations were statistically significant (P<0.001).

**Table 4:** A Passing-Bablok regression analysis for the two analyzers. CI: confidence intervals

Parameter	Regression equation	Intercept A (95%CI)	Slope B (95%CI)
CK	y = 0.769 + 1.077 x	0.769 (-0.027 to 1.333)	1.077 (1.067 to 1.087)
LDH-1	y = -0.206 + 0.8504 x	-0.206 (-1.579 to 0.775)	0.851 (0.838 to 0.868)
RBP	y = -4.351 + 0.947 x	-4.351 (-4.960 to -3.705)	0.947 (0.928 to 0.966)
Cys-C	y = -0.023 + 0.653 x	-0.023 (-0.050 to 0.003)	0.653 (0.628 to 0.678)
IgA	y = 0.092 + 0.925 x	0.092 (0.066 to 0.118)	0.925 (0.914 to 0.936)
IgM	y = 0.044 + 0.995 x	0.044 (0.029 to 0.056)	0.995 (0.981 to 1.011)
IgG	y = -0.238 + 0.840 x	-0.238 (-0.600 to 0.115)	0.840 (0.808 to 0.871)

**Figure 1:**
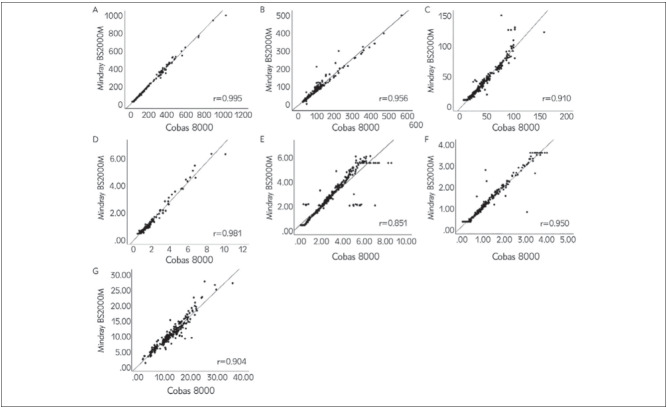
Spearman rank correlation of evaluated parameter between the two machines (A) CK, (B) LDH-1 (C) RBP, (D) Cys-C, (E) IgA, (F) IgM, (G) IgG.

On the Bland-Altman plot, the average bias in Cys-C, IgA, and IgM was close to zero (0.520, 0.189, and 0.046, respectively), while the average bias of CK and LDH-1 in the two machines were -11.938 and 12.180, respectively (Table 3). In particular, the comparison of Cobas 8000 and Mindray data showed a significant negative bias for CK while the bias was positive for LDH-1 and RBP (Figure 1). In addition, three-sevenths of two instruments had more than 95% of the points within the 95% consistency limit (RBP 96.4%, IgM 95.6%, and IgG 95.0%) in Bland-Altman analysis, meeting the consistency requirements. The remaining four items were also more than 90% (data not shown). The absolute value of the difference between the two machines was less than 10% which demonstrates that the difference is clinically acceptable.

Figure 2

**Figure 2:**
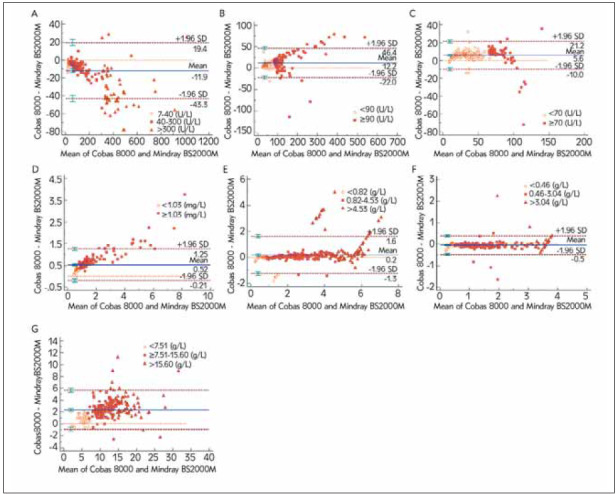
Bland-Altman plots of the difference of the evaluated parameter between the two machines (A) CK, (B) LDH-1 (C) RBP, (D) Cys-C, (E) IgA, (F) IgM, (G) IgG.

Figure 3

**Figure 3:**
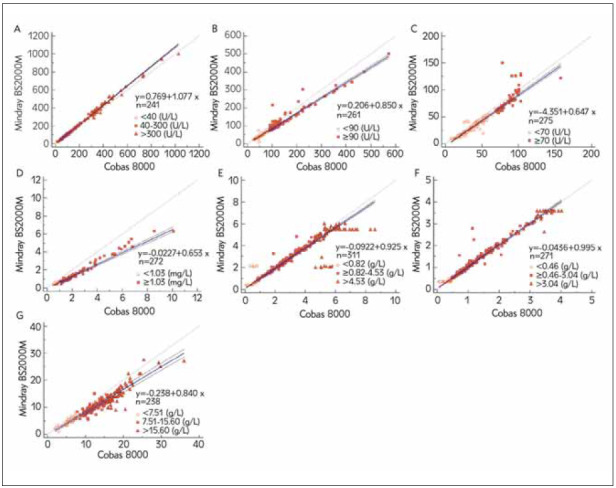
Passing-Bablok regression of the difference of the evaluated parameter between the two machines (A) CK, (B) LDH-1 (C) RBP, (D) Cys-C, (E) IgA, (F) IgM, (G) IgG.

According to Passing and Bablok regression analysis, 95% CI for the intercept A of the regression equation for CK, LDH-1, Cys-C, and IgG includes 0, and there is no systematic error between the two instruments. For IgA and IgM, the 95% CI for intercept A was very close to zero. Only a relatively high intercept A can be found in RBP (intercept A: -4.351, 95% CI -4.960 to -3.705). Except for IgM, the 95% CI of the slope B contains 1 (0.9806-1.0105), another the slope B of CK, RBP, and IgA were almost equal to 1 (1.077, 0.947, and 0.925, respectively). For LDH-1, Cys-C, and IgG, slope B did not contain 1 (0.851, 0.653, and 0.840, respectively), which shows some proportional differences between the two instruments (Table 4). Therefore, it can be considered that the results of the two pieces of equipment are consistent, and the two devices are interchangeable.

## Discussion

The availability of rapid and automated methods regarded as a major breakthrough in the laboratory can decrease the labor force and increase consistency and repeatability. Indeed, in addition to improving the clinical effectiveness, the new generation of automated analyzers increases laboratory efficiency by reducing working time and costs associated with the optical validation of the results. At present, the most regularly used chemistry platforms in the laboratory are Abbott, Beckman Coulter, Roche Cobas, and Mindray. Different detection systems using different methods will produce different results for different samples on different detected platforms, and this difference may affect routine clinical decision-making. Hence, when utilizing different analyzers to disclose the same items, the instrument needs to be contrasted with guaranteeing the consistency and conformity of the detected results. Numerable studies have focused on comparing biomarkers in Abbott, Hitachi, and Roche [6] [14]. As previously described in the literature, these clinical chemistry assays are accurate and reliable and are readily applicable on various platforms. Some newly launched and advanced chemical and immune analyzers remain uncertain. This study aimed to compare basic biochemistry parameters between Roche Cobas 8000 and Mindray BS2000M.

To our knowledge, this is the first large study using two automated chemistry platforms Roche Cobas 8000 and Mindray BS2000M, to assess the equivalence of common organ function parameters. A total of 1869 samples were screened in our study. The ultimate objective was to evaluate whether the detected values in different analyzers were identical and therefore interchangeable when informing clinicians' decisions in diagnosis, treatment, and prognosis. All items in the two platforms were appraised according to CLSI protocols [7] [25].

In the assessment of linearity, the r for all analytes at all levels was more than 0.9 except for IgA (*r*=0.851). All items within the clinical reference range showed excellent linearity. However, the linearity of RBP, Cys-C, IgA, and IgM at a low or high level was verified outside the range as claimed by the manufacturer. Regarding the correlation of parameters in two systems, we found that the correlation of all analytes at all levels was highly relevant (CC> 0.95, *P*<0.001). However, the CC of IgA and IgM at a high level showed a low correlation (0.605 and 0695, respectively). According to the regression equation between Roche Cobas 8000 and Mindray BS2000M, CK, IgA, and IgM performance were excellent in our study, which did not show a statistically significant proportional error or constant error. On the contrary, RBP in the two instruments displayed a significant constant error (intercept A=-4.351), and Cys-C showed obviously proportional error (slope B=0.653). There remained a small proportional error in LDH-1 (slope B=0.851) and IgG (slope B=0.840).

In Bland-Altman's plot, Cys-C, IgA, IgM, RBP, and IgG showed a low average bias (0.520, 0.189, 0.046, 5.579, and 2.354, respectively), and their mean bias in the former three almost closed to 0. While for CK and LDH-1, the mean differences were higher (-11.938 and 12.180, respectively) and the same as the limit of agreement (LOA), proportionally increasing with the growing levels (CK: -43.303 to 19.428, LDH-1: -22.020 to 46.380). For instance, their average bias showed significant differences in CK (-3.846 to -33.041) and LDH-1 (7.288 to 16.677), compared with the Cys-C, IgA, and IgM. We suggest three possible explanations for why the average bias of CK and LDH-1 was so wide. One possible reason is that the two platforms use the different detection methods for CK (colorimetry vs. creatine phosphate substrate method) and LDH-1 (rate method vs. lactic acid substrate method). The study of He et al. demonstrated that the coefficient of variation of Cys-C showed a significant difference (P=0.016), very low pass rates, and widespread distributions (from 3.63% to 6.74%) in internal quality control of laboratories using different systems from 2014 to 2017 in China [29]. Meanwhile, Han et al. [30] study also showed that LDH-1 should be improved their precision and accuracy at the same time after being evaluated sigma index, further supporting our investigation. Another factor caused by the significant mean difference was that the detection limits of different platforms are different. If the true value of parameters exceeds upper detection limits, one of the common solutions in regular work of laboratories to solve high-level samples is for an operator to dilute the sample by adding low level serum or matrix [31]. A previous study also demonstrated that substrate depletion plays a key role in causing negative results. The enzyme linearity extension function in BS-2000M2 can effectively solve the risk of false-negative results for high-level samples [32]. Hence, to avoid unnecessary misleading and misconceptions, the sample from one patient should not be detected separately on different methods of different systems in the same laboratory. One should not use sample internal quality control rule if it is necessary to use a different sample to verify or review the values of parameter. Moreover, it is wise and advisable for different laboratories to establish reference ranges and dilute high levels samples beyond upper limitation.

This study has mentioned limitations. One of them was that the performance of our study only compared with two analyzers (Roche Cobas 8000 and Mindray BS2000M) and did not include more clinical chemistry platforms, such as Abbott and Hitachi. Due to the small volume of samples, there was no possibility of repeating the analysis with every analyzer once more. Another disadvantage was that the samples included in our study contained all kinds of patients and healthy people. Further study on the performance of biochemical or immune items by various analyzers in a more significant number of cases and multicenter should be performed to validate the findings of this study. Based on the data in our study, we can conclude that the analytical performances of RBP, Cys-C, IgA, IgM, and IgG are excellent, while CK and LDH-1 need to be improved to decrease or remove the systematic error as much as possible.

Taken together and to the best of our knowledge, this is the first study to describe the performance characteristics of the Roche Cobas 8000 and Mindray BS2000M systems. The two platforms have good correlation and bias for detecting CK, LDH-1, RBP, Cys-C, IgM, and IgG analytes. They have a high method agreement in CK, LDH-1, IgA, IgM, and IgG. In summary, Cobas and Mindray clinical chemistry assays are reliable and precise, and applicable to different analytic platforms.

## Dodatak

### Acknowledgments

This research was funded by Key Laboratory of Early Prevention and Treatment for Regional High- Incidence-Tumor, Guangxi Medical University, Ministry of Education (GKE2019-05, GKEZZ202132).

### Conflict of interest statement

All the authors declare that they have no conflict of interest in this work.

### List of abbreviations

CLSI, Clinical and Laboratory Standards Institute;<br>CK, creatine kinase;<br>AMI, acute myocardial infarction;<br>LDH-1, lactate dehydrogenase-1;<br>RBP, retinol-binding protein;<br>Cys-C, Cystatin-C;<br>IgA, immunoglobulin A;<br>IgM, immunoglobulin M;<br>IgG, immunoglobulin G;<bR>CSF, cerebrospinal fluid;<br>NABL, the National Laboratory Accreditation Board;<br>CI, confidence interval;<br>CV, coefficient of variation;<br>LOA, the limit of agreement;<br>Min, minimum;<br>Max, maximum;<br>CC, correlation coefficient.
